# Frontiers in hepatic drug delivery-grand challenges

**DOI:** 10.3389/fddev.2023.1265446

**Published:** 2023-10-20

**Authors:** José M. Lanao

**Affiliations:** Pharmaceutical Sciences Department, Area of Pharmacy and Pharmaceutical Technology, Institute for Biomedical Research of Salamanca (IBSAL), University of Salamanca, Salamanca, Spain

**Keywords:** hepatic drug delivery, gene delivery, cell-based therapies, microfluidics, 3D printing, PBPK-PD modeling, machine learning, hepatic toxicity

## Introduction

Currently, there is a wide range of liver diseases with health and socioeconomic impact. The high incidence of chronic liver diseases is estimated to impact 1.5 billion people and two million deaths around the world (Moon et al., 2020).

The development of hepatic drug and gene delivery systems represents an important challenge for researchers, considering cellular target identification and the cell signalling complex associated with liver diseases such as liver fibrosis, different types of hepatitis, fatty liver disease, or non-alcoholic fatty liver diseases (NAFLD), among others. Other important challenges that must be considered in this field are the use of cell-based therapies or the new methodologies of manufacturing. In addition, the *in vitro—in-silico* approaches, the application of PBPK-PD modeling and simulation and the artificial intelligence methodologies already used in drugs with liver effects as well as advancements in the knowledge of the hepatic toxicity of nanosystems are also important challenges.

## Search for therapeutic targets

In liver injury, liver-resident cells such as hepatocytes, liver sinusoidal endothelial cells, activated hepatic stellate cells (HSCs) and, activated kupffer cells or innate and adaptive immune cells may play different roles in the pathogenesis and disease resolution of different liver diseases such as liver fibrosis, hepatitis or hepatocellular carcinoma and are potential targets for drug therapy (van der Heide et al., 2019; Colino et al., 2020; Gong et al., 2023). Moreover, in liver pathogenesis, knowledge of liver cells’ intercellular communication is essential, as well as the carrier role of extracellular vesicles of miRNA used as biomarkers in the diagnosis of different liver diseases (Sato et al., 2019).

Liver diseases are mediated by different types of cellular receptors that are differently expressed in liver cells mRNA forms of the damaged liver and which are specific molecular targets for different types of therapies (Mishra et al., 2022).

The understanding of the mechanisms involved in liver pathogenesis and the identification of specific cellular receptors as liver targets constitutes an important challenge for the development of new drug and gene delivery systems.

## Drug and gene delivery systems innovations

In the last years, there has been significant progress in nanosystems development using different types of nanoparticles, mainly lipids, to target drugs and genetic material to the diseased liver for the diagnosis and treatment of liver diseases. The use of functionalized nanomaterials for selective targeting should be highlighted. Gene therapy is also a commitment to the future and progress has been made in the treatment of liver diseases such as hepatocellular carcinoma or liver fibrosis, among others (Mahdinloo et al., 2020; Mahmoud et al., 2022).

Targeting hepatocytes by using different nanosystems with drugs or genetic material such as nanoemulsions, liposomes, self-emulsifying DDS, micelles, polymeric nanoparticles or nanogels, among others, has been tested for the treatment of nonalcoholic fatty liver disease (NAFLD) (Kotsiliti, 2023).

Different types of lipid-based, polymeric, and inorganic nanoparticles containing drugs such as Imatinib, Nilotinib and genetic material such as SiRNA were developed to target Hepatic Stellate Cells in the diagnosis or treatment of liver fibrosis (Bai and Zhai, 2020).

Considering that hepatic macrophages play an important role in the progression of liver diseases, different drug delivery systems targeting hepatic macrophages have been proposed, such as PLGA nanoparticles, liposomes, or micelles, among others, with immunomodulatory, anti-inflammatory, or antifibrotic activity (Van der Heyde et al., 2019; Colino CI et al., 2020).

For the treatment of hepatitis B (HBV), different types of lipid, polymeric or conjugate nanoparticles containing anti-HBV nucleoside drugs or genetic material such as Ribozyme or RNAi were developed. Also, gene editing technology (CRISPR/Cas) mediated by nanoparticles as well as vaccines against HBV has been introduced (Miao et al., 2021). Progress has also been made in the use of different types of nanoparticles with antiviral drugs and vaccines for the treatment of hepatitis C (HBC) (Elberry et al., 2017).

Liver cancer is one of the areas of greatest progress in the development of delivery systems. They must ensure selective distribution in the tumour, drug release, and system stability (Yang et al., 2022). Advanced therapies in this field also include immunotherapy and gene therapy based on the use of nanoparticles, mainly liposomes, and viral vectors (Chakraborty and Sarkar, 2022). Also, drug-eluting bead transarterial chemoembolization based on the use of microspheres has been proposed as an alternative for the treatment of hepatocellular carcinoma (Li et al., 2023).

Different types of delivery systems containing chemotherapeutic drugs or gene therapy are based on the use of different kinds of nanoparticles, functionalized nanoparticles, or stimulus-responsive nanocarriers (Graur et al., 2022; Mahmoud et al., 2022; Radu et al., 2022). Important advances are also being made in the diagnosis of liver cancer using different types of nanosystems, such as gold nanoparticles, magnetic nanoparticles, or functionalized nanoparticles with fluorescent probes, among others (Chowdhury et al., 2021).

CRISPR/Cas gene editing techniques associated with drug delivery systems to target genetic material in liver cancer should be highlighted due to the important role they will play soon (Chakraborty and Sarkar, 2022).

## Advancements in cell-based therapies

An important challenge in hepatic drug delivery is the use of therapies based on cells and cell-derived vehicles. Different types of cells such as hepatocytes, mesenchymal, embryonic or stem cells, and ghost erythrocytes have been tested in experimental studies and clinical trials for the treatment of chronic liver diseases, mainly against liver failure and liver fibrosis. In recent years, Car-T cell and TCR therapies have had a big impact on cancer immunotherapy. The use of non-viral vectors such as lipid nanoparticles for mRNA delivery in primary human T cells has been proposed to produce CAR T cells for cancer tumour targeting (Billingsley et al., 2020; Yuan et al., 2021; Li TT et al., 2022).

Dendritic cells are leucocytes involved in innate and adaptive immunity and can be used as drug-delivery systems in autoimmune and inflammatory diseases. The use of this type of cell may be an alternative to nanoparticles to deliver mRNA for cancer immunotherapy applications or other pathologies (Firdessa-Fitte R and Creusot, 2019).

In recent years there has been significant progress in the use of exosomes obtained from liver cells and other sources as cell-based therapy strategies to treat liver cancer or non-alcoholic fatty liver diseases, among others, using drugs and genetic material (Gutierrez-Millán et al., 2021; Ding et al., 2023).

Although progress has been made in the field of cell-based therapies, important issues such as cell administration, dosage and duration of treatment, manufacturing at scale and regulatory pathways must be optimized (Li et al., 2022).

## New technologies

Organ on a chip constitutes a promising technology with important applications in the development of drug delivery systems. In recent years there has been an evolution from two-dimensional (2D) cell cultures such as hepatocytes to three-dimensional (3D) cell culture models, spheroids, and organ-on-a-chip devices. Despite the limitations to reproducing liver pathologies *in vitro* compared to models in experimental animals, this type of system presents future possibilities in the evaluation of hepatic drug delivery systems (van Grunsven, 2017; Deng et al., 2019; Leedale et al., 2020; Monteduro et al., 2023).

On the other hand, future cell transplantation strategies have been proposed to repair tissue in damaged liver using biomaterial-based cell delivery with natural or synthetic materials. Biomaterials may be combined with biomolecules or cells such as primary hepatocytes, stem cells or exosomes. These engineered biomaterial-based scaffolds with/without spheroids may be manufactured using innovative methods such as microfluidics, 3D-printing, electrospinning, or freeze drying, among others, these must guarantee their biocompatibility and their activity, allowing hepatic cell proliferation (Da Silva Morais et al., 2020; Kim et al., 2021; Rahmati et al., 2023). This type of drug or genetic material-loaded scaffold, could also be used to control drug release into the liver tissue (Kumar et al., 2019).

In the last years, microfluidics and nanofluidics have emerged as revolutionary manufacturing technologies with implications in drug delivery, showing advantages such as dose optimization, multiple dosing, controlled release, low-cost manufacturing, and good functionality, among others (Da Silva Morais et al., 2020; Naveen et al., 2022). Additive manufacturing such as 3D and 4D bioprinting is also a technology with a great future projection and with important biomedical applications, especially in regenerative medicine and tissue engineering (E.g., Spheroids manufacturing) (Banerjee et al., 2022).

At the regulatory level, different agencies have published different guidelines and technical documents related to the development, manufacturing, quality control, or preclinical studies of nanomedicines, mainly liposomes. The FDA and EMA guidances about nanomaterials, focus on the targeting capacity of specific tissues such as the liver, as well as on the hepatic mechanisms of elimination. Likewise, the FDA guidance affects the need to increase information regarding the interactions of nanomaterials with biological systems. On the other hand, regulatory aspects, especially at the clinical level, are still limited (EMA guidance, 2019; FDA guidance, 2022; Liu et al., 2022).

## In silico approaches, modeling and simulation methodologies

*In vitro—In-silico* optimization based on computer simulations of nanosystems physicochemical and biopharmaceutical properties allows us to characterize drug-polymer and other material interactions as well as the drug loading capacity and drug release (Bouzo et al., 2020).

Physiologically based Pharmacokinetic-Pharmacodynamic (PBPK-PD) modeling and simulation is an interesting methodology to predict biodistribution, target interaction, and pharmacological response and toxicity of delivery systems, playing an important role in dose optimization. Considering the physiological complexity of the liver, this type of modeling allows simulation of the physiological changes in the liver associated with liver disease and its incidence on the pharmacokinetic and pharmacodynamic behaviour of drugs, at the preclinical and clinical level, incorporated into different types of delivery systems (Colino et al., 2020).

One of the current challenges in biomedical research is the application of artificial intelligence tools for the diagnosis and treatment of liver diseases. In this framework, machine learning technologies based on artificial neural networks and other methods will be used soon for the development and optimization of hepatic drug delivery systems. Drug delivery issues such as the selection of nanomaterials, process optimization, controlled release, dose calculation, or clinical trial design, among others, may be addressed through this type of methodology (Villa Nova et al., 2022).

## Assessment of nanomaterials hepatic toxicity

Different toxic effects on the liver and other organs have been associated with various nanomaterials, such as silica nanoparticles, metallic nanoparticles, or carbon nanotubes, among others. Most of the nanoparticles that enter the systemic circulation are eliminated through the reticuloendothelial system, increasing the research interest in materials hepatic toxicity, which represents a significant challenge.

Toxicokinetics is an emerging science that contributes to knowledge about the toxicity and clearance of nanomaterials as well as the mechanisms and factors involved that are not sufficiently clarified, especially in the diseased liver (Sun et al., 2021).

Looking ahead, further research on nanomaterials liver toxicity as well as the mechanisms involved is needed. Therefore, the increasing use of physiologically based toxicokinetic-toxicodynamic models (PBTK_TD) will have special relevance, allowing the toxicity of nanosystems to be related to the liver disease’s physiological and pathological factors (Sun et al., 2021; Phatruengdet et al., 2022).

## Progress in clinical trials

In recent years, various clinical trials have been carried out in different phases using drug and gene delivery systems against liver diseases. For example, in hepatocellular carcinoma, different clinical trials were performed with doxorubicin, gemcitabine, mitoxantrone, aroplatin, and irinotecan-type chemotherapeutics and siRNA oligonucleotide, double-stranded RNA, or microRNA-type genetic material. Diverse systems were used, such as liposomes, pegylated liposomes, thermally sensitive liposomes, polymers, or glycyrrhizin conjugates, using passive and active targeting strategies and stimulus-responsive release (Chakraborty and Sarkar, 2022).

Clinical trials are underway with polymeric nanoparticles of PEG-Interferon Alfa combined with Tenofovir Disoproxil Fumarate in Hepatitis B related liver fibrosis, PEGylated fibroblast growth factor 21 in NASH and liver cirrhosis or PEG-Intron plus Rebetol treatment in chronic HCV with liver fibrosis (Vyas and Patel, 2023).

In addition, non-viral gene therapy clinical trials were carried out using siRNA incorporated into lipid nanoparticles against different therapeutic targets such as amyloidosis, hepatitis B, liver cancer, hepatocellular carcinoma, atherosclerosis or liver fibrosis associated with NASH or hepatitis C (Witzigmann et al., 2020; Vyas and Patel, 2023). Also, gene therapy clinical trials for anti-HBV using non-viral vectors were performed (Miao et al., 2021). Regarding cell-based therapies, there is very limited clinical experience and clinical trials should be strengthened. Table 1 shows some examples of clinical trials on the use of hepatic drug and gene delivery systems for the treatment of different liver pathologies (https://www.clinicaltrials.gov/).

**TABLE 1:** Examples of some completed or active clinical trials of drug and gene delivery systems (non-viral vectors) including cell-based therapies for hepatic drug delivery (https://www.clinicaltrials.gov/).

Identifier	Status	Phase/Study	Delivery system	Intervention/Treatment	Conditions
NCT02181075	Completed	Phase 1	Lyso-thermosensitive Liposomal Doxorubicin	Drug: ThermoDox^®^ (LTLD)	Liver tumour
				Device: Focused Ultrasound	
NCT00093444	Completed	Phase 1	Liposomal doxorrubicin	Drug: lyso-thermosensitive liposomal doxorubicin	Liver cancer, metastatic cancer
				Procedure: radiofrequency ablation	
NCT00869778	Completed	Phase 2	liposome-based nanoparticle lipopeptide vaccine	Biological: εPA-44	Chronic hepatitis B
NCT05900050	Recruiting	Phase 2	liposomal formulation VS-01	Drug: VS-01 on top of SOC	Acute on Chronic liver failure, ascites
				Other: SOC (Control Group)	
NCT05039632	Recruiting	Phase 1-2	crystalline hafnium oxide (HfO2) functionalised nanoparticles	Other: Hafnium Oxide-containing Nanoparticles NBTXR3	Advanced malignant solid neoplasm, metastatic malignant neoplasm in the liver, others
				Radiation: Radiation Therapy	
NCT00441376	Completed	Phase 1	Thermally Sensitive Liposomal Doxorubicin	Drug: ThermoDox	Hepatocellular carcinoma, liver neoplasms
NCT00019630	Completed	Phase 1	Doxorubicin HCl Liposomes	Drug: doxorubicin HCl liposome	Childhood liver cancer, others
NCT00293397	Completed	Not applicable	Doxorubicin microspheres	Device: Drug-eluting beads loaded with doxorubicin hydrochloride	Liver cancer
NCT02112656	Completed	Phase 3	thermally sensitive liposomal doxorubicin	Drug: ThermoDox	Hepatocellular carcinoma
				Drug: Dummy infusion	
NCT00617981	Completed	Phase 3	thermally sensitive liposomal doxorubicin	Drug: ThermoDox	Hepatocellular carcinoma
				Drug: 5% Dextrose Solution	
NCT01004978	Active	Phase 3	LC bead	Drug: Cisplatin, Doxorubicin, LC bead, Mitomycin, Sorafenib, Placebo Administration	Hepatocellular carcinoma, unresectable hepatocellular carcinoma
NCT05093920	Active	Early Phase 1	Doxorubicin microspheres	Drug: Doxorubicin-Eluting Beads	Hepatocellular carcinoma
NCT03706157	Completed	Phase 4	Lipiodol-based emulsions	chemoembolization with doxorubicin	Hepatocellular carcinoma
NCT01655693	Completed	Phase 3	Doxorrubicin nanoparticles	Drug: Doxorubicin	Hepatocellular carcinoma
NCT05519475	Recruiting	Phase 2	siRNA nanoparticles	Drug: ALN-HSD, placebo	Nonalcoholic steatohepatitis
NCT02191878	Completed	Phase 1-2	Si-RNA lipid nanoparticles	Drug: TKM-080301	Hepatocellular carcinoma. Liver cancer, others
NCT02227459	Completed	Phase 1	Si-RNA lipid nanoparticles	Drug: ND-L02-s0201 Injection	Moderate to extensive hepatic fibrosis
NCT04682847	Active	Observational	Iron Oxide Nanoparticles (SPION) on MRI-Linac	Drug: Ferumoxytol injection	Liver neoplasms, hepatic cirrhosis, hepatic carcinoma liver cancer, others
NCT01437007	Completed	Phase 1	Si-RNA lipid nanoparticles	Drug: TKM-080301	Different types of cancer with hepatic metastases
NCT02716012	Active	Phase 1	RNA liposomal nanoparticles	Drug: MTL-CEBPA	Hepatocellular carcinoma, liver cancer
				Drug: Sorafenib 200 mg	
NCT05497453	Recruiting	Phase 1-2	mRNA lipid nanoparticles	Drug: OTX-2002, Tyrosine kinase inhibitor	Hepatocellular carcinoma, liver cancer, others
NCT05560607	Recruiting	Phase 1	mRNA lipid nanoparticles	Drug: AZD7503 Intervention	Non-Alcoholic Fatty Liver Disease (NAFLD)
NCT04745403	Recruiting	Phase 1	HBV-TCR redirected T-cells	Drug: mRNA HBV/TCR T-cells	Hepatocellular carcinoma
NCT05123209	Recruiting	Phase 1	IM83 CAR-T cells	Biological: IM83 CAR-T cells	Liver cancer
				Combination Product: second-line treatment of liver cancer	
NCT05195294	Not yet recruiting	Phase 1-2	HBV Antigen-specific TCR	Biological: LioCyx-M	Hepatocellular carcinoma liver cancer, adult
				Drug: Lenvatinib	liver cell carcinoma
NCT05905731	Active	Phase 1	Autologous T cells	Biological: TCR-T	Chronic hepatitis B
NCT02686372	Completed	Phase 1	Redirected T Cell	Biological: TCR-T	Hepatocellular carcinoma
				Biological: No intervention	

Despite these and other advancements, it will be necessary to increase progress in clinical trials of new hepatic drug and gene delivery systems that allow the generation of marketed products.

## Conclusion

In summary, the main challenge shortly will be the overall integration of new knowledge on specific targets in hepatic diseases, advanced experimental methods based on new manufacturing methodologies and functionalized biomaterials, drug *in-vitro in-vivo* approaches based on in-silico models, PBPK modeling and simulation and artificial intelligence. All this will allow to develop innovative drug and gene delivery systems, including cell-based therapies. On the other hand, it is essential to increase the number of clinical trials with these new systems, as well as the assessment of the regulatory implications.
